# Proteome Exploration to Provide a Resource for the Investigation of *Ganoderma lucidum*

**DOI:** 10.1371/journal.pone.0119439

**Published:** 2015-03-10

**Authors:** Guo-Jun Yu, Ya-Lin Yin, Wen-Hui Yu, Wei Liu, Yan-Xia Jin, Alok Shrestha, Qing Yang, Xiang-Dong Ye, Hui Sun

**Affiliations:** 1 State Key Laboratory of Virology, College of Life Sciences, Wuhan University, Wuhan, China; 2 Key Laboratory of Combinatorial Biosynthesis and Drug Discovery (Ministry of Education), Wuhan University, Wuhan, China; 3 Hubei Provincial Cooperative Innovation Center of Industrial Fermentation, Key Laboratory of Fermentation Engineering (Ministry of Education), Hubei University of Technology, Wuhan, China; Henan Agricultural Univerisity, CHINA

## Abstract

*Ganoderma lucidum* is a basidiomycete white rot fungus that has been used for medicinal purposes worldwide. Although information concerning its genome and transcriptome has recently been reported, relatively little information is available for *G*. *lucidum* at the proteomic level. In this study, protein fractions from *G*. *lucidum* at three developmental stages (16-day mycelia, and fruiting bodies at 60 and 90 days) were prepared and subjected to LC-MS/MS analysis. A search against the *G*. *lucidum* genome database identified 803 proteins. Among these proteins, 61 lignocellulose degrading proteins were detected, most of which (49 proteins) were found in the 90-day fruiting bodies. Fourteen TCA-cycle related proteins, 17 peptidases, two argonaute-like proteins, and two immunomodulatory proteins were also detected. A majority (470) of the 803 proteins had GO annotations and were classified into 36 GO terms, with “binding”, “catalytic activity”, and “hydrolase activity” having high percentages. Additionally, 357 out of the 803 proteins were assigned to at least one COG functional category and grouped into 22 COG classifications. Based on the results from the proteomic and sequence alignment analyses, a potentially new immunomodulatory protein (GL18769) was expressed and shown to have high immunomodulatory activity. In this study, proteomic and biochemical analyses of *G*. *lucidum* were performed for the first time, revealing that proteins from this fungus can play significant bioactive roles and providing a new foundation for the further functional investigations that this fungus merits.

## Introduction

*Ganoderma lucidum* is a basidiomycete white rot fungus and has been one of the best-known medicinal macrofungi for many years [1, 2]. This fungus’s pharmacological activities are widely recognized, as evidenced by its inclusion in the American Herbal Pharmacopoeia and Therapeutic Compendium [2–7]. Many different types of active ingredients have been identified (e.g., polysaccharides, triterpenoids, and lignocellulose degrading enzymes), indicating that this mushroom is a cellular reservoir for biologically useful compounds [8–16].

With the rapid development of next-generation sequencing technology [17, 18], the genome and transcriptome of *G*. *lucidum* of different strains and at different developmental stages have recently been sequenced [19–24]. By analyzing the *G*. *lucidum* genome, 417 genes have been assigned to carbohydrate-active enzyme (CAZymes) families [19, 25]. However, the actual existence of these lignocellulose degrading enzymes has yet to be experimentally verified by, for example, mass spectrometry sequencing technology [26–28].

After genomics, proteomics is thought to be the next most powerful approach for the study of complex biological systems [29, 30]. The recent evolution of rapid protein identification technologies has made large-scale proteome analysis possible [31]. One of the most common methods in proteomic analysis is the use of one-dimensional or two-dimensional gel electrophoresis (1-DGE or 2-DGE) followed by enzymatic hydrolysis and mass spectrometry analysis [32–38]. Using this method, several proteomic studies have been conducted with fungi and mushrooms, including *Termitomyces heimii* [39], *Sparassis crispa* [35], *Hericium erinaceum* [35], *Arthrobotrys oligospora* [40], *Metarhizium acridum* [30], Agrocybe aegerita [41] and *Cordyceps militaris* [42]. These studies provided a useful informational resource for proteins. However, because comparable genomic information for these organisms was lacking at the time that the proteomic investigations were done, the proteomic investigations were mostly based on the non-redundant protein database of the National Center for Biotechnology Information (NCBInr).

Many proteins in macrofungi are bioactive. The examples include Fip-vvo from *Volvariella volvacea* [43], GMI from *Ganoderma microsporum* [44, 45], IPAF from *Anoectochilus formosanus* [46], AAL and AAL-2 from *Agrocybe aegerita* [47, 48], and immunomodulatory protein LZ-8 from *G*. *lucidum* [49, 50]. Considering the availability of *G*. *lucidum* genome, we assume there should be many undiscovered proteins in *G*. *lucidum* which remain to be identified by proteomic methods. Although several secretome proteins were analyzed [51], and a class of natural glycopeptides with sugar moiety-dependent antioxidant activities was published [52], comprehensive proteomic studies were still missing for *G*. *lucidum*.

In this study, we report the first proteomic characterization of *G*. *lucidum*. A total of 803 proteins were identified from *G*. *lucidum* by LC-MS/MS. Many significant proteins were detected, such as lignocellulose degrading proteins, tricarboxylic acid cycle related proteins, peptidases, argonaute-like proteins, and immunomodulatory proteins. In addition, based on the results of proteomic analysis, we cloned and expressed a new immunomodulatory protein named GL18769, which exhibited high immunomodulating activity.

## Materials and Methods

### Sources of *G*. *lucidum*

**Fig 1:**
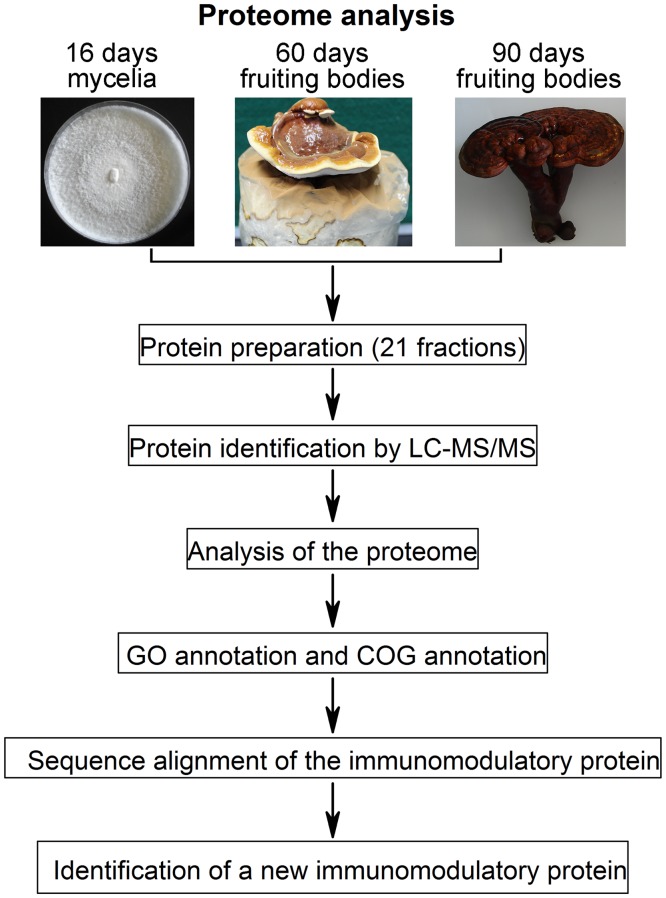
Analysis pipeline of the G. lucidum proteome.

The 16-day mycelia and fruiting bodies (at 60, 90 days) of *G*. *lucidum* (yw-1 strain) were obtained from the Guangdong Institute of Microbiology (Guangdong, China, Fig. 1). As described previously [21], vegetative mycelia were grown on potato dextrose agar plates in the dark at 25°C, and the fruiting bodies were cultured on basswood medium bags (from Guangdong Institute of Microbiology). Three independent samples at each growth stage were used for this study.

### Preparation of protein samples from *G*. *lucidum* at three developmental stages

Proteins were prepared from 16-day mycelia (16dM) and 60-day fruiting bodies (60dF) using the same method, and the procedure is as follows. Fresh mycelia (1 g) or 60dF (5 g) were ground into a fine powder with the use of liquid nitrogen. A protein mixture was obtained using the trichloroacetic acid/acetone (TCA/acetone) method as previously described [53]. Briefly, 10 ml of cold TCA/acetone solution (10% TCA (w/v) and 0.07% β-mercaptoethanol in acetone) was added to 1 g of fungal powder, and the suspension was thoroughly vortexed for 1 h at -20°C and were centrifuged at 16,000 × g for 15 min at 4°C. The supernatant was discarded and the pellet was washed three times with pre-chilled washing solution (0.07% β-ME, 2 mM EDTA, and EDTA-free proteinase inhibitor cocktail tablets (Roche) in 100% acetone) followed by the removal of all the residual acetone. The pellet was dried and solubilized in 100 μl homogenization buffer (0.2 M Tris-HCl buffer, pH 7.8, containing 5 mM EDTA·2Na, 14 mM β-ME, 10% (v/v) glycerol and 2 EDTA-free proteinase inhibitor tablets (Roche) per 100 ml of buffer solution in MQ H_2_O). To solubilize the protein pellet further, 60 μl SDS-sample buffer (2.5×, 62 mM Tris (pH 6.8) containing 10% (v/v) glycerol, 2.5% (w/v) SDS, and 5% (v/v) 2-ME, pH 6.8) was added to the mixture, followed by vortexing and sonication. After centrifugation at 16,000×g for 10 min at 4°C, the supernatant was collected and subjected to protein quantification using the Pierce BCA method.

**Fig 2:**
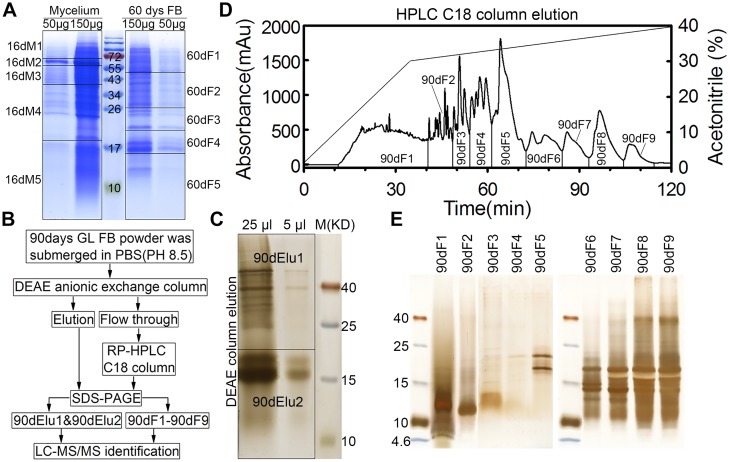
Protein preparation from three different developmental stages of *G*. *lucidum*. (A) Protein preparation of mycelium (16 days) and fruiting bodies at 60 days (60dF). The grids indicate how the SDS-PAGE gel bands (16dM1–16dM5, 60dF1–60dF5) were cut for MS identification. The middle lane represents the molecular weight of the markers (kDa). (B) Workflow of protein separation from fruiting bodies at 90 days (90dF). (C) SDS-PAGE of the DEAE column elution fractions of the 90dF total proteins. The gel was silver stained and prepared as two fractions (90dElu1 and 90dElu2) before mass spectrometry. (D) The DEAE flow through fraction of total proteins from 90dF (90dF1–90dF9) was separated by HPLC. The x-axis represents the run time of HPLC method, the left y-axis shows the absorbance value of proteins at 280 nm and the right y-axis indicates the acetonitrile concentration. (E) The 9 HPLC fractions were dialyzed, lyophilized and subjected to SDS-PAGE and silver staining.

Approximately 100 g of the 90-day fruiting bodies (90dF) were crushed into a fine powder and extracted twice with 1.5 L cold 0.01 M PBS (pH 8.5) and 10 EDTA-free proteinase inhibitor cocktail tablets at 4°C for 24 hours. The supernatant was collected by centrifugation at 12,000 × g for 20 min at 4°C and loaded onto a DEAE Sepharose Fast Flow (GE Healthcare) column equilibrated with 10 mM PBS (Fig. 2B). The bound materials were eluted with the same buffer containing 1 M NaCl. Both the flow through fraction and the eluate were collected. The flow through fraction was further separated by reverse phase high-performance liquid chromatography (RP-HPLC) using an RP-HPLC column (Flexar, PerkinElmer, C_18_ column, 10 × 250 mm). The elution was carried out with a 0% to 30% gradient of acetonitrile in 0.1% (v/v) trifluoroacetic acid (TFA) at 2 ml/min for 35 min, and then with a 30% to 40% gradient of acetonitrile in 0.1% TFA at 0.8 ml/min for 85 min. Nine fractions were collected (Fig. 2D), and each was dialyzed extensively against distilled water and lyophilized. The DEAE column eluate (with 1 mM NaCl) and nine HPLC fractions were separately concentrated by the TCA/acetone method described above.

### One-Dimensional Gel Electrophoresis (1-DGE) and Mass Spectrometry Analysis

The proteins prepared from the 16-day mycelium and the 60dF (200 μg per sample) were separated on a 15% SDS-PAGE gel. The gel was stained with Coomassie brilliant blue (CBB) R-250, and divided into 10 sections (Fig. 2A). For the 90dF, the protein pellet of the DEAE eluate, originating from 100 g of 90dF, was dissolved in 500 μl of SDS-PAGE loading buffer, from which 30 μl was taken and resolved on a 15% SDS-PAGE gel. The gel was stained with a SilverSNAP Stain Kit II (PIERCE, Thermo scientific) and divided into 2 sections (Fig. 2C). Each of the 9 HPLC fractions was dissolved in 100 μl of SDS-PAGE loading buffer and 10 μl was applied to a 15% SDS-PAGE gel. The gel was stained with a SilverSNAP Stain Kit II (Fig. 2E). These 21 gel slices were ‘in-gel’ reduced, S-alkylated and digested with trypsin [42]. The tryptic peptides derived from the gel bands were subsequently separated by a C_18_ reverse-phase column and analyzed on a nanoelectrospray ionization mass spectrometer (microTOF-Q II ESI-Q-ToF LC/MS/MS, Bruker Daltonics, Germany) operated in the positive ion mode. After sample loading and desalting at 4 μl/min, a Switchos II column switching device transferred the flow paths to the analytical column. The nanoflow was eluted at 400 nl/min using a 90 min gradient from 90% solvent A (0.1% formic acid in H_2_O) to 90% solvent B (0.1% formic acid in ACN). The ESI-MS was operated in a data-dependent MS/MS mode in which each full MS scan was followed by five MS/MS scans. The nanospray voltage was 1.5 kV and the MS data acquisition time was set to 3 s per spectrum over a m/z range of 300–1500 Da.

The MS/MS data were processed further by Flex Analysis software (Bruker Daltronics) using the recommended parameters (mass window for precursor ion selection: 2; relative collision energy: 27%; parameters for dynamic exclusion: 15 seconds). Protein identification was performed by searching against a virtual protein database (16,495 sequences) translated from the *G*. *lucidum* genome using the Mascot program (http://www.matrixscience.com). The search parameters were set to 15 ppm and 0.6 Da for peptide and fragment mass tolerance, respectively. The fixed modification was carbamidomethyl (C) and the variable modification was Gln->pyro-Glu (N-term Q), Oxidation (M). For individual data analysis, the significance threshold p < 0.05 and the Mascot score ≥ 25 were considered to be the standards for assigning a positive match to a protein in the database. The false discovery rates (FDR) were tested for all experimental runs using the Decoy option in Mascot and were 1%, both at the peptide and protein levels (FDR < 0.01).

### Functional annotations of the proteome

The proteins identified by LC-MS/MS were searched against the functional annotation database of *G*. *lucidum* genome [19]. GO annotation and COG annotation of our proteome were extracted from the functional annotation database of *G*. *lucidum* and subjected to classification using the GO (http://geneontology.org/) and COG websites (http://www.ncbi.nlm.nih.gov/COG/), respectively [54, 55].

### Sequence analysis of immunomodulatory proteins

The amino acid sequences of 11 published immunomodulatory proteins (gi|636613877, gi|597978919, gi|636613749, gi|729544, gi|597981577, gi|283488736, gi|597978931, gi|187961980, gi|126657, gi|348167218 and gi|62739082) were downloaded from NCBI website. The sequences of GL18769 and GL18770 were extracted from the *G*. *lucidum* genome data [19]. The sequence alignment of these 13 proteins was performed using ClustalX and Jalview software.

### Cloning and expression of GL18769 gene in *Escherichia coli*

Total RNA from the 60-day fruiting bodies of *G*. *lucidum* was extracted using TriZol reagent (Promega). Full-length cDNAs were synthesized from 1 μg of total RNA using MMLV (Moloney murine leukaemia virus) reverse transcriptase (Promega) after RQ1 RNase-free DNase treatment (Promega) according to the manufacturer’s instructions. According to the current proteomic study and the published *G*. *lucidum* genome data, the nucleotide sequence of GL18769 was extracted from *G*. *lucidum* genome [19]. To clone the GL18769 coding sequence, the following primer pair was used: sense primer, 5'-CATGCCATGGATGCCCTCCAACACCGCTCT-3'; anti-sense primer, 5'-CCCAAGCTTGTTCCACTGGGCGATGAGGT-3'. PCR was performed using KOD-Plus-Neo (KOD-401) DNA polymerase (TOYOBO) with the following temperature profile: 5 min at 94°C, 20 s at 94°C, 30 s at 55°C, 11 s at 68°C and 5 min at 68°C, for 28 cycles. The PCR product was isolated and cloned into pET-28a vector (Novagen). The resulting plasmid containing the GL18769 coding sequence was transformed into *E*. *coli* BL21(DE3) cells (TransGen Biotech, Lot#G301130). Induction of protein expression was performed with 0.5 mM IPTG (isopropylβ-D-thiogalactopyranoside) at the mid-exponential phase (*D*_*600*_ of 0.4–0.6), and the bacteria were grown for an additional 4 h at 37°C on a shaker at 220 rev/min. The bacteria were harvested and lysed, and the supernatant of the lysate was loaded on to a His Trap FF column (GE Healthcare) using ÄKTAprime plus (General Electric Company). The GL18769 protein was eluted with elution buffer (20 mM sodium phosphate, 0.5 M NaCl, 500 mM imidazole, pH 7.4) at a flow rate of 1 ml/min and detecting the absorbance at 280 nm.

### Blast-formation stimulatory activity

Six-week-old male C57BL/6 mice were purchased from Wuhan University Center for Animal Experiment/Animal Biosafety Level III Lab (A3Lab) and housed in a specific pyrogen-free room until used in the experiment. All study protocols were approved by the Institutional Animal Care and Use Committee (IACUC) of the Wuhan University School of Medicine (Wuhan, China) in accordance with the regulations of the National Institute of Health “Guide for the Care and Use of Laboratory Animals” and all details of animal welfare and steps taken to ameliorate suffering were in accordance with the recommendations of the Weatherall report. Three mice were killed by cervical dislocation and their spleens were aseptically removed. The spleen cells were collected by passage through a wire screen (300 mesh) using EZ-Sep Mouse 1 x Lymphocyte Separation Medium (Dakewe Biotech Co., Ltd.) according to the manufacturer’s protocol. The red blood cells in the cell suspension were hemolyzed with 0.17 M Tris-HC1 (pH 7.7) containing 0.16M NH_4_Cl. After washing the cells with 15 ml of RPMI 1640 and centrifuging at 800 × g for 10 min, the cells were re-suspended in 8 ml of RPMI 1640 supplemented with 10% fetal calf serum, 100 units/ml penicillin, and 100 pg/ml streptomycin. The cell density was then adjusted to 3 × 10^6^/ml. Splenic lymphocytes (0.1 ml, 3 × 10^5^ cells/well) were seeded onto a 96-well microtiter plate and incubated with various concentrations of GL18769 protein (2.5, 5, 10 μg/ml) or with Concanavalin A (ConA, 2 μg/ml). The cells were maintained at 37°C under 5% CO_2_ in air for 36 hours, and 10 μl of CCK-8 was then added to the cells (Cell Counting Kit-8, Dojindo, http://dojindo.cn/products/C/cck-8.htm), followed by an additional incubation for 1–2 h. The absorbance at 450 nm was measured for each well and the ‘relative fold change’ of the treated condition was calculated by following the manufacturer’s protocol [56]. The cell morphology was also monitored microscopically (LEICA DM IRB) at a 50 X magnification.

### Statistical analysis

The two-sample Student’s *t* test was used for comparisons between groups of the "Blast-formation Stimulatory Activity" assay. Statistical analysis was performed using GraphPad Prism 5 and Origin 7 software. The results were expressed as means ± S.E.M., and the statistical significance was defined as P<0.05.

## Results and Discussion

### Preparation of protein from *G*. *lucidum*

Fig. 1 presents the workflow outline for the *G*. *lucidum* proteome analysis carried out in this study. *G*. *lucidum* is a macrofungus which undergoes tremendous changes during the developmental stages from mycelium to mature fruiting body (Fig. 1). To study the proteome of *G*. *lucidum* comprehensively and identify as many proteins as possible, proteins from three different developmental stages of *G*. *lucidum* were extracted. Based on the previous studies on *G*. *lucidum* [19, 21], we chose *G*. *lucidum* mycelia at approximately 16 days and fruiting bodies at approximately 60 days. In addition, because fruiting bodies at approximately 90 days have been widely used as a traditional herbal medicine and the commercial products are readily available from the market [1], we also chose the 90-day *G*. *lucidum* an experimental material.

The proteins of 16-day mycelia (16dM) and 60-day fruiting bodies (60dF) were extracted by the same TCA/acetone method and subsequently resolved by SDS-PAGE (Fig. 2A). As expected, the protein profiles were different between 16dM and 60dF, especially in the regions corresponding to 17–26 and 43–72 kDa. The gels were cut horizontally into 5 slices (16dM1–16dM5, 60dF1–60dF5) for each sample.

Because 90-day fruiting bodies (90dF) of *G*. *lucidum* are highly lignified, we needed to use a different method to extract the proteins (see Materials and methods). The protein mixtures extracted by PBS (pH 8.5) were further separated by DEAE anion-exchange and HPLC reverse-phase column chromatography (Fig. 2B). The eluate from the DEAE column was resolved by SDS-PAGE and the gel was cut into two slices (90dElu1 and 90dElu2) (Fig. 2C). The DEAE flow through was loaded onto a HPLC C_18_ column and separated into 9 fractions (90dF1–90dF9) (Fig. 2D), which were then subjected to SDS-PAGE followed by silver staining (Fig. 2E). Compared with the protein profiles of 16-day mycelium and the 60-day fruiting bodies, most proteins of the 90-day fruiting body were distributed in the 4.6–40kDa region, especially in the 10–25 kDa area (Fig. 2C and Fig. 2E).

In total, 21 protein fractions (16dM1–16dM5, 60dF1–60dF5, 90dElu1, 90dElu2 and 90dF1–90dF9) from three different developmental stages of *G*. *lucidum* were produced for the following MS identification.

### Proteomic characterization of *G*. *lucidum*

**Fig 3:**
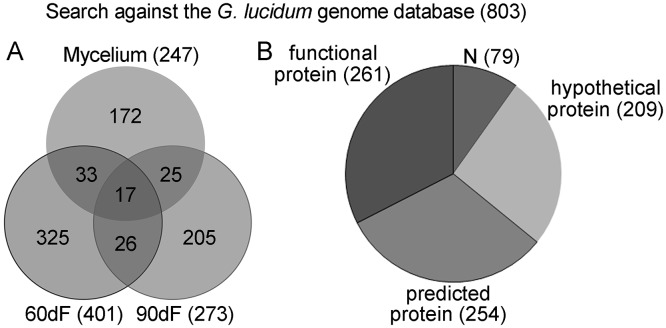
Proteomic results from combined SDS-PAGE/LC-MS/MS analysis. (A) Venn diagrams of proteomes from the three developmental stages of *G*. *lucidum*. By searching against genome database, 803 proteins were identified with 247, 401 and 273 proteins detected from 16dM, 60dF and 90dF stages, respectively. (B) Distribution of the 803 proteins identified. Among the 803 proteins, 79, 209, 254 and 261 proteins were grouped into ‘N’, ‘hypothetical protein’, ‘predicted protein’ and ‘functional protein’ classes, respectively. ‘N’ represents proteins with no particular annotations, while ‘functional protein’ represents proteins that have a clear functional annotation.

The 21 protein fractions were digested with trypsin and subjected to proteomic characterization by LC-MS/MS. The peptides identified by mass spectrometry were analyzed using the Mascot program and searched against the *G*. *lucidum* genome database. A total of 803 non-redundant proteins was identified from all the samples combined (S1 Table, FDR < 0.01 for protein identifications). Fig. 3A shows the distribution of proteins among the different developmental stages, as revealed by SDS-PAGE and LC-MS/MS analysis. Respectively, 247, 401 and 273 non-redundant proteins were detected from the samples at 16dM, 60dF and 90dF stages. Among these proteins, 17 were found in samples from all three stages, including an immunomodulatory protein Ling Zhi-8 (GL18770), α-galactosidase (GL30909), exo-1,3-β-glucanase (GL30087), translation elongation factor (GL29943), glycoside hydrolase (GL29873 and GL23600), aspergillopepsin (GL26523), and glutathione reductase (GL22863). Respectively, 172, 325 and 205 proteins could only be found in 16dM, 60dF and 90dF samples (Fig. 3A).

Compared to human and model organisms, functional proteome studies of non-model organisms, including macroscopic fungi, are not as advanced. Thus among the 803 proteins identified in the *G*. *lucidum* genome, 79 proteins were defined as ‘N’ (no annotation), 209 as ‘hypothetical protein’, and 254 as ‘predicted protein’ (Fig. 3B and S1 Table); only 261 out of 803 proteins had particular functions annotated. Although the remaining 542 proteins had no specific functional annotations available at this time, the current proteomic analysis at least confirmed the physical existence of these proteins in *G*. *lucidum*, enriching the protein database for this fungus. Further investigation should be performed to discover the function of those proteins not annotated to date.

#### 1. Identification of wood-degrading enzymes

At present, the limitation of fossil fuel reserves and its negative impact on the environment have spurred an urgent need for the development of alternative energy resources that could meet future demands. The wood-degrading enzyme family has been considered as a valuable resource because of its potential to produce sustainable biofuels from lignocellulose substrates. We detected 61 lignocellulose degrading proteins with high MS scores in the *G*. *lucidum* proteome (Table 1). These proteins included catalase, laccase, cellobiose dehydrogenase, endoglucanase, cellobiohydrolase, alginate lyase, chitinase, carbohydrate esterase, glycoside hydrolase, and exo-1,3-beta-glucanase (Table 1). Searching against the CAZy database [25, 57–59] allowed the classification of these 61 wood degrading enzymes into the families of Auxiliary Activities (AAs, 15 proteins), Carbohydrate-Binding Modules (CBMs, 13), Carbohydrate Esterases (CEs, 3), Glycoside Hydrolases (GHs, 28), GlycosylTransferases (GTs, 1) and Polysaccharide Lyases (PLs, 1). Many of them (28 proteins) were thus grouped into the GHs family. The relative abundance of GHs enzymes observed in our proteome is consistent with a previous genomic study of *G*. *lucidum* which showed that this fungus contained the largest number of GH enzymes among the six CAZy families (GHs, CEs, GTs, PLs, CBAs and AAs) [19]. The detailed sub-categories of the 61 proteins are shown in Table 1. Among the 61 lignocellulose degrading proteins, 15, 13, and 49 proteins were found in the extracts from mycelium, 60dF, and 90dF, respectively, indicating that the fruiting bodies at 90 days may contain more related enzymes (Table 1).

**Table 1:** Detailed information of 61 identified lignocellulose degrading proteins.

Protein ID^a^	Protein Description	Protein MW(Da)	Mascot score	Protein coverge(%)	Matched peptides^b^	CAZy families^c^	Stage^d^
GL22189-R1_1	catalase	59605	15218	48.1	18	AA1	16dM,60dF
GL16398-R1_1	laccase	56868	118	7.3	2	AA1	90dF
GL21497-R1_1	laccase	59332	37	2.8	1	AA1	90dF
GL22256-R1_1	cellobiose dehydrogenase	84146	25	6.9	1	AA3	60dF
GL25348-R1_1	copper radical oxidase	70424	51	5.6	1	AA5	16dM
GL27858-R1_1	copper radical oxidase	100390	108	7.7	3	AA5	90dF
GL21192-R1_1	copper radical oxidase variant A	82336	423	17.6	5	AA5	90dF
GL24805-R1_1	glucooligosaccharide oxidase	53907	366	17.6	6	AA7	90dF
GL24807-R1_1	glucooligosaccharide oxidase	59940	582	24.2	9	AA7	90dF
GL23615-R1_1	glucooligosaccharide oxidase	35311	165	18.2	2	AA7	90dF
GL24786-R1_1	glucooligosaccharide oxidase	57385	111	7.9	1	AA7	90dF
GL24789-R1_1	glucooligosaccharide oxidase	58094	28	8.8	2	AA7	90dF
GL30499-R1_1	cytochrome b2	54647	32	11.8	1	AA8	16dM
GL28634-R1_1	endo-beta-glucanase	32410	209	16.2	3	AA9	60dF,90dF
GL24196-R1_1	endoglucanase	41497	91	5.4	2	AA9	90dF
GL18725-R1_1	cellobiohydrolase I	50008	709	37.6	10	CBM1	90dF
GL29727-R1_1	cellobiohydrolase I	51017	466	26.1	7	CBM1	90dF
GL30351-R1_1	cellobiohydrolase I	50138	212	18.8	7	CBM1	90dF
GL24712-R1_1	cellobiohydrolase II	47213	237	16.3	4	CBM1	90dF
GL26036-R1_1	endo-1,4-beta-xylanase C precursor	50246	108	7.4	2	CBM1	90dF
GL20229-R1_1	endo-1,4-B-xylanase A	44982	539	29.6	9	CBM1	90dF
GL25283-R1_1	mannanase	49168	36	3.7	1	CBM1	90dF
GL16814-R1_1	carbohydrate-binding module family 12 protein	35744	538	10.6	1	CBM12	16dM
GL22047-R1_1	carbohydrate-binding module family 13 protein	15848	116	33.8	2	CBM13	16dM,90dF
GL16341-R1_1	alginate lyase	33672	168	11.9	3	CBM2	90dF
GL25627-R1_1	chitinase	46320	62	3.5	1	CBM2	90dF
GL30108-R1_1	carbohydrate binding domain-containing protein from family CBM21	86164	28	1.5	1	CBM21	16dM
GL21331-R1_1	putative laminarinase	35134	35	3.8	1	CBM4	90dF
GL26613-R1_1	candidate lipase/esterase from carbohydrate esterase family CE10	93064	34	3.1	1	CE10	16dM
GL29877-R1_1	candidate polysaccharide deacetylase from carbohydrate esterase family CE10	54773	204	3.8	1	CE10	60dF
GL28882-R1_1	carbohydrate esterase family 15 protein	42513	73	7.3	2	CE15	90dF
GL24039-R1_1	candidate beta-glucosidase from glycoside hydrolase family 1	55862	46	3.7	1	GH1	16dM
GL30087-R1_1	exo-1,3-beta-glucanase	47029	174	12.5	4	GH132	16dM,60dF,90dF
GL23395-R1_1	exo-beta-1,3-glucanase	82884	95	2.6	1	GH132	90dF
GL31059-R1_1	glycoside hydrolase family 13 protein	59698	516	19.5	8	GH132	90dF
GL23580-R1_1	glycoside hydrolase family 15 protein	61201	185	20.2	4	GH15	60dF,90dF
GL23600-R1_1	glycoside hydrolase family 15 protein	57540	347	16.5	5	GH15	16dM,60dF,90dF
GL25075-R1_1	glycoside hydrolase family 16 protein	71204	127	5.5	2	GH16	90dF
GL24376-R1_1	glycoside hydrolase family 18 protein	67342	43	1	1	GH18	90dF
GL21024-R1_1	glycoside hydrolase family 27 protein	34228	55	4.2	1	GH27	90dF
GL27011-R1_1	glycoside hydrolase family 27 protein	19926	56	6.3	1	GH27	90dF
GL20947-R1_1	endo-polygalacturonase PG1	37820	204	22.7	3	GH28	90dF
GL19093-R1_1	beta-xylosidase	88017	85	4.6	3	GH3	60dF,90dF
GL22886-R1_1	beta-xylosidase	87234	70	5.9	3	GH3	90dF
GL27550-R1_1	glycoside hydrolase family 3 protein	85637	58	4.8	3	GH3	90dF
GL21973-R1_1	endo-arabinase	30804	28	1.8	1	GH43	90dF
GL15164-R1_1	glycoside hydrolase family 43 protein	35013	69	14.3	2	GH43	90dF
GL15780-R1_1	glycoside hydrolase family 43 protein	36021	100	13	3	GH43	90dF
GL20698-R1_1	glycoside hydrolase family 47 protein	59518	808	26.8	9	GH47	90dF
GL21451-R1_1	glycoside hydrolase family 55 protein	93018	397	12.8	7	GH55	90dF
GL29873-R1_1	glycoside hydrolase family 72 protein	94656	243	4.2	3	GH72	16dM,60dF,90dF
GL30540-R1_1	glycoside hydrolase family 74 protein	76162	73	5.4	2	GH74	90dF
GL26459-R1_1	glycoside hydrolase family 79 protein	53522	92	6.4	1	GH79	16dM,90dF
GL29728-R1_1	D-xylose reductase	35040	1202	38.3	6	GH8	16dM,60dF
GL18249-R1_1	glycoside hydrolase family 92 protein	93316	53	10.9	2	GH92	60dF,90dF
GL23422-R1_1	glycoside hydrolase family 92 protein	93686	181	10	4	GH92	16dM,90dF
GL29257-R1_1	glycoside hydrolase family 92 protein	78244	120	1.4	1	GH92	60dF
GL29258-R1_1	glycoside hydrolase family 92 protein	84221	252	19.3	7	GH92	90dF
GL21099-R1_1	glycoside hydrolase family 95 protein	90612	507	16	10	GH95	60dF,90dF
GL21375-R1_1	glycogen phosphorylase	114415	2759	16.5	11	GT35	16dM
GL23979-R1_1	polysaccharide lyase family 8 protein	79492	186	8.5	3	PL8	90dF

^a^, matched protein ID was derived from the *G*. *lucidum* genome database.

^b^, the details of matched peptides was shown in S1 Table.

^c^, these wood-degrading enzymes were classified into CAZy sub-families.

^d^, the identified proteins were from three developmental stages of *G*. *lucidum* (16dM, 60dF, 90dF).

#### 2. Characterization of other proteins

In addition to the 61 identified wood degrading proteins, other important proteins were also characterized (Table 2). Fourteen proteins are involved in the tricarboxylic acid (TCA) cycle [60, 61], including aconitate hydratase [62], citrate synthase [63], fumarate reductase [64], glyceraldehyde-3-phosphate dehydrogenase, phosphoglucomutase [65], phosphopyruvate hydratase, pyruvate kinase [66], succinate semialdehyde dehydrogenase [67], succinate-CoA ligase, acetyl CoA carboxylase, glucose-6-phosphate 1-dehydrogenase, and mannose-6-phosphatase. Most of these proteins (10) were found in the 16-day mycelium (Table 2), suggesting the significance of TCA cycle during *G*. *lucidum* development. Two N-acetylhexosaminidases and one UTP-glucose-1-phosphate uridylyltransferase were found [68, 69]. These three proteins are involved in the hexosamine biosynthesis pathway [70]. Of 17 different peptidases detected in the proteome [71, 72], most (12) were found in the fruiting bodies at 90 days. This probably explains the SDS-PAGE protein profile of 90dF (see above) in which most protein bands occurred in the 10–25 kDa region (Fig. 2C and Fig. 2E). Aflatoxin-detoxifizyme is a protein that detoxifies aflatoxins [73]. Aldehyde dehydrogenase plays an important role in acetaldehyde detoxification [74]. Argonaute-like protein is involved in the expression of miRNA-like RNAs in fungi [75]. The identification of two argonaute-like proteins suggests the potential existence of miRNA-like RNAs in *G*. *lucidum*. Farnesyl-diphosphate synthase and beta-glucan synthesis-associated proteins are involved in triterpenoid biosynthesis and polysaccharide biosynthesis, respectively [76, 77]. Two immunomodulatory proteins (GL18770 and GL18769) were also detected from the proteome of this medicinal fungus.

**Table 2:** A list of proteins involved in tricarboxylic acid cycle, hexosamine biosynthesis pathway, peptidase and other interesting bioactivities.

Protein ID	Protein Description	Protein MW(Da)	Mascot score	Protein coverge(%)	Matched peptides^a^	Stage
**Tricarboxylic acid cycle**						
GL21959-R1_1	acetyl CoA carboxylase	252793	61	2.5	1	16dM
GL18572-R1_1	aconitate hydratase	85821	54	8.1	2	16dM
GL24555-R1_1	citrate synthase	51487	30	3.3	1	90dF
GL20259-R1_1	citrate synthase	55510	35	2.2	1	16dM
GL17816-R1_1	fumarate reductase	67459	77	11.9	5	90dF
GL31587-R1_1	glucose-6-phosphate 1-dehydrogenase	56070	299	9.1	2	16dM
GL21313-R1_1	glyceraldehyde-3-phosphate dehydrogenase	34528	1910	22.3	4	60dF
GL20532-R1_1	mannose-6-phosphatase	37754	436	32.8	10	16dM,90dF
GL24763-R1_1	mannose-6-phosphatase	39883	114	7.4	2	90dF
GL24280-R1_1	phosphoglucomutase	61444	554	15.9	5	16dM,60dF
GL30114-R1_1	phosphopyruvate hydratase	46944	9340	69.5	18	16dM,60dF
GL30680-R1_1	pyruvate kinase	61232	8782	47.2	18	16dM
GL23356-R1_1	succinate semialdehyde dehydrogenase	53519	102	9.4	2	16dM
GL29894-R1_1	succinate-CoA ligase	44637	27	5.5	1	16dM
**Hexosamine biothesis pathway**						
GL24346-R1_1	N-acetylhexosaminidase	60350	200	10.8	5	16dM,90dF
GL24347-R1_1	N-acetylhexosaminidase	81223	192	8.3	4	90dF
GL25739-R1_1	UTP-glucose-1-phosphate uridylyltransferase	58901	255	27.8	5	16dM
**Peptidase**						
GL18792-R1_1	aspartic peptidase A1	47176	190	8.5	3	60dF,90dF
GL19589-R1_1	aspartic peptidase A1	45789	110	8.3	2	90dF
GL26523-R1_1	aspergillopepsin	27828	1104	4.5	1	16dM,60dF,90dF
GL18287-R1_1	aspergillopepsin A	43958	1784	15.4	2	16dM,60dF
GL23283-R1_1	endopeptidase	94366	37	5.5	1	16dM,60dF
GL31420-R1_1	endopeptidase	46824	2238	34.4	11	16dM
GL28218-R1_1	metallopeptidase MepB	86674	719	12.4	5	16dM
GL20779-R1_1	peptidase M28	52988	349	18.5	5	90dF
GL24319-R1_1	peptidyl-Lys metalloendopeptidase	37080	95	19.3	3	90dF
GL24399-R1_1	peptidyl-Lys metalloendopeptidase	38070	128	13.5	2	90dF
GL24396-R1_1	Peptidyl-Lys metalloendopeptidase	38694	432	12.7	2	90dF
GL24402-R1_1	Peptidyl-Lys metalloendopeptidase	36457	25	3.5	1	90dF
GL24406-R1_1	Peptidyl-Lys metalloendopeptidase	36648	28	8.5	1	90dF
GL24410-R1_1	Peptidyl-Lys metalloendopeptidase	13937	62	9.4	1	90dF
GL26638-R1_1	serine carboxypeptidase	53487	66	2.6	1	90dF
GL29874-R1_1	serine carboxypeptidase	56049	53	3.3	1	90dF
GL31396-R1_1	tripeptidyl peptidase A	66494	28	7	1	60dF
**Other interesting proteins**						
GL31548-R1_1	aflatoxin-detoxifizyme	77614	472	4.3	2	16dM,60dF
GL30174-R1_1	aldehyde dehydrogenase	58137	5806	32.9	12	16dM
GL15827-R1_1	argonaute-like protein	112897	31	1.2	1	90dF
GL31293-R1_1	argonaute-like protein	79522	108	8.8	1	60dF
GL29980-R1_1	beta-glucan synthesis-associated protein	118511	55	1.2	1	90dF
GL25499-R1_1	farnesyl-diphosphate synthase	41202	66	3.3	1	16dM
GL23374-R1_1	high nitrogen upregulated cytochrome P450 monooxygenase 2	65111	30	5.9	1	60dF
GL24810-R1_1	high nitrogen upregulated cytochrome P450 monooxygenase 2	56857	45	2	1	60dF
GL18770-R1_1	immunomodulatory protein	17501	828	11	1	16dM,60dF,90dF
GL18769-R1_1	immunomodulatory protein	12534	682	12.5	1	60dF,90dF
GL25550-R1_1	manganese peroxidase	38810	115	2.5	1	16dM
GL15091-R1_1	PAH-inducible cytochrome P450 monooxygenase PC-PAH 3	63420	160	2.7	1	16dM
GL28943-R1_1	PAH-inducible cytochrome P450 monooxygenase PC-PAH 4	59857	35	2.8	1	16dM
GL20529-R1_1	superoxide dismutase	27197	3190	18.1	3	16dM,90dF

^a^, the details of matched peptides was shown in S1 Table.

In summary, the 803 proteins identified by this study confirmed the existence of many putative proteins predicted from the *G*. *lucidum* genome and thus greatly enriched the *G*. *lucidum* protein database.

### Functional annotation of the *G*. *lucidum* proteome

As shown in Fig. 1, to analyze the *G*. *lucidum* proteome further, the 803 non-redundant proteins were subjected to annotation by GO and COG [54, 55].

#### 1. Gene ontology annotation

**Fig 4:**
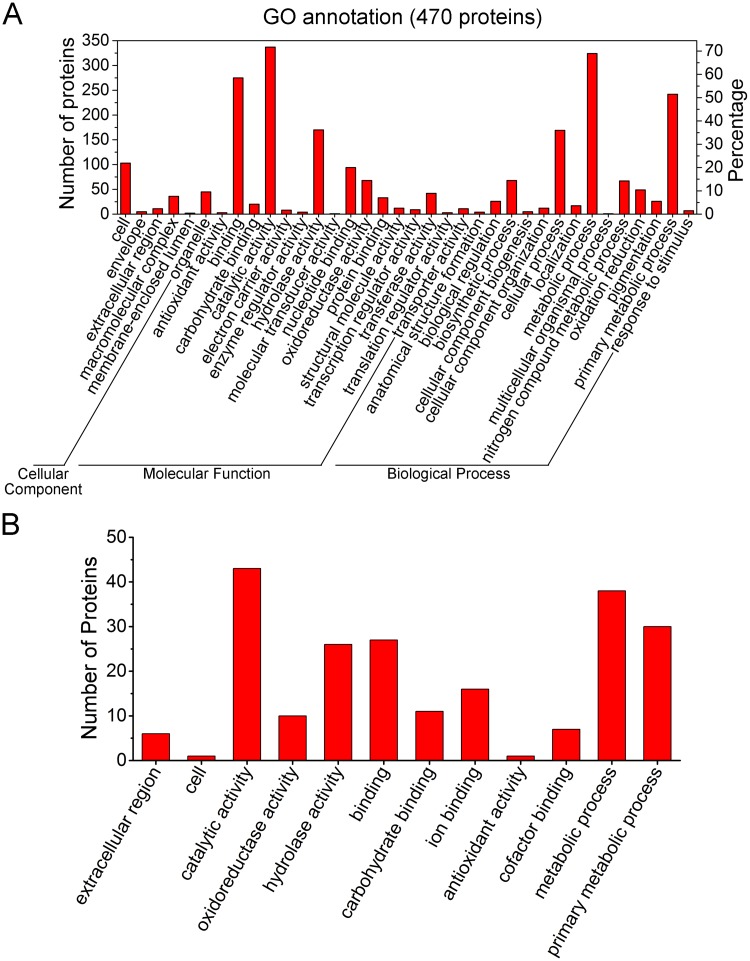
GO annotation of the *G*. *lucidum* proteome. (A) GO annotation of the total identified 803 proteins. GO annotations for 470 proteins were classified into 36 GO sub-categories. The results were summarized in three main GO categories: cellular component, molecular function and biological process. The left y-axis indicates the number of proteins in a sub-category. The right y-axis indicates the percentage of a specific sub-category of proteins in that main category. (B) GO annotation of the 61 wood-degrading proteins. GO annotations for 43 out of the 61 proteins were classified into 12 GO sub-categories. One protein could be annotated into more than one GO term.

A total of 470 proteins were annotated across the GO sub-categories (S2 Table) and classified into 36 functional groups with 6 involved in cellular component, 16 in molecular function and 14 in biological processe (Fig. 4A). Among these GO classifications, ‘binding’ (275 proteins, 58.5%), ‘catalytic activity’ (337, 71.7%), ‘hydrolase activity’ (170, 36.2%), ‘cellular process’ (169, 36%), ‘metabolic process’ (324, 68.9%), and ‘primary metabolic process’ (242, 51.5) were dominant. In contrast, a small number of proteins were assigned to ‘membrane-enclosed lumen’ (2 proteins, 0.4%), ‘molecular transducer activity’ (1, 0.2%), and ‘multicellular organismal process’ (1, 0.2%).

Among the 61 identified wood degrading proteins, 43 of them received annotations across GO sub-categories, grouped into 12 functional groups (Fig. 4B). Among these GO categories, ‘catalytic activity’ (43 proteins, 100%), ‘hydrolase activity’ (26, 60.5%), ‘binding’ (27, 72.8%), ‘metabolic process’ (38, 88.4%), and ‘primary metabolic process’ (30, 69.8%) were dominant. All of these GO categories were related to the lignocellulose degradation, suggesting that the current proteome analysis is reasonably accurate.

#### 2. COG annotation

**Fig 5:**
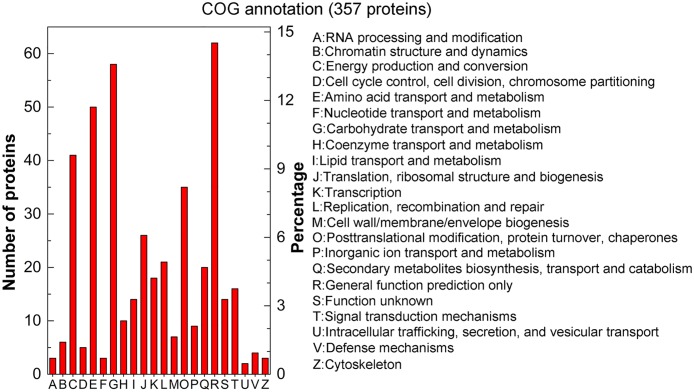
COG annotation of the total identified 803 proteins. COG annotations for 357 proteins were grouped into 22 categories. The left y-axis indicates the number of proteins in a particular category. The right y-axis indicates the percentage of a specific category of proteins in that main category.

To further evaluate the effectiveness of our annotation process and the accuracy of our proteome analysis, we searched the identified proteins against COG classifications. A total of 357 proteins were assigned to at least one COG functional category, grouped into 22 classifications (Fig. 5 and S3 Table). Of the 22 COG categories, ‘General function prediction only’ (14.52%) contained the most proteins, followed by ‘Carbohydrate transport and metabolism’ (13.58%), ‘Amino acid transport and metabolism’ (11.71%), and ‘Energy production and conversion’ (9.6%). These COG classifications are consistent with the results described above (Table 1) in which many wood-degrading proteins were detected in our proteome. A few proteins related to ‘Intracellular trafficking, secretion, and vesicular transport’ (0.47%), ‘Cytoskeleton’ (0.7%), ‘Nucleotide transport and metabolism’ (0.7%), and ‘RNA processing and modification’ (0.7%) were found. These annotations provide new resources for further exploring this macrofungus.

### Cloning and expression of the gene for a new immunomodulatory protein (GL18769)

**Fig 6:**
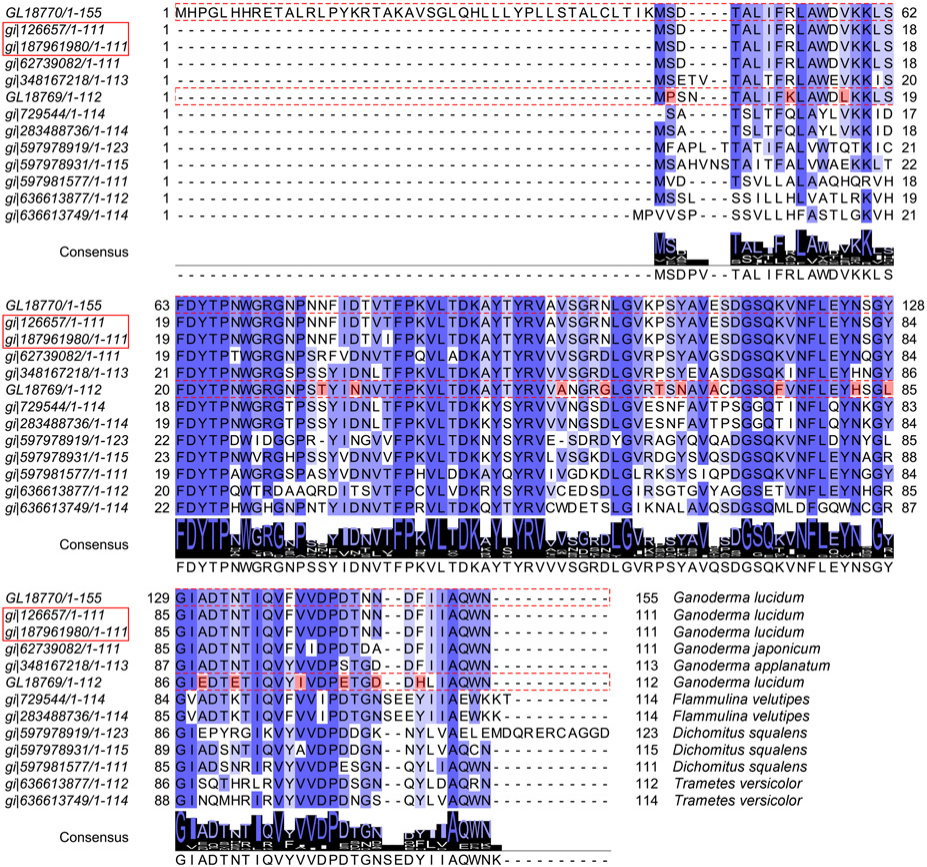
Sequence analysis of 13 immunomodulatory proteins. GL18770 and GL18769 were identified from the *G*. *lucidum* proteome. The immunomodulatory protein sequences of *Ganoderma lucidum* (gi|126657 and gi|187961980), *Ganoderma japonicum* (gi|62739082), *Ganoderma applanatum* (gi|348167218), *Flammulina velutipes* (gi|283488736 and gi|729544), *Dichomitus squalens* (gi|597981577, gi|597978931 and gi|597978931) and *Trametes versicolor* (gi|636613877 and gi|636613749) were all available on the NCBI website. The protein ID in the red rectangle represents the LZ-8 protein ID. Strictly conserved residues are indicated by blue shadows and are also displayed in the ‘Consensus’ sequence. The residues in red shadow are the significant amino acid differences of GL18769 from the other proteins. GL18770 had an identical amino acid sequence to LZ-8protein (gi|126657 and gi|187961980), except for the redundant 44 amino acids at its N-terminus. GL18769 had a 72.32% identity to LZ-8 protein.

Previous studies reported the existence of immunomodulating activities in *G*. *lucidum* [3, 4, 6, 49]. Many studies have been published on immunomodulatory proteins, indicating the pharmacological significance of this activity [49, 78–80]. From the *G*. *lucidum* proteomic analysis, two immunomodulatory proteins were identified in the fruiting bodies at both 60 and 90 days (Table 2). Previous studies reported that fungal immunomodulatory proteins (FIPs) belonged to a new protein family with high sequence and structural similarities [81]. Therefore, we downloaded 11 different immunomodulatory proteins from the NCBI website and performed a sequence alignment between them and the two proteins, GL18769 and GL18770, which we identified as likely immunomodulatory proteins (Fig. 6) from our proteome. Both GL18769 and GL18770 showed a high similarity with the 11 proteins from *Ganoderma lucidum* (gi|126657 and gi|187961980), *Ganoderma japonicum* (gi|62739082), *Ganoderma applanatum* (gi|348167218), *Flammulina velutipes* (gi|283488736 and gi|729544), *Dichomitus squalens* (gi|597981577, gi|597978931 and gi|597978931), and *Trametes versicolor* (gi|636613877 and gi|636613749). From this alignment, we could see that these proteins shared the same conserved amino acid sequence. Moreover, the recognition of this consensus sequence could aid in immunomodulatory protein engineering in the future (Fig. 6).

Except for a redundant 44 amino acid sequence at the N-terminus, GL18770 is essentially identical to LZ-8 protein (gi|126657 and gi|187961980). GL18769, on the other hand, only has a 72.32% similarity to LZ-8 (gi|126657 and gi|187961980) (Fig. 6). GL18770 is the LZ-8 protein and the minor differences between them might result from different *G*. *lucidum* strains used in the respective studies. Nevertheless, as shown in Fig. 6, GL18769 had 19 amino acid differences from the other immunomodulatory proteins, suggesting that GL18769 could be a new immunomodulatory protein. To assess the reliability of our proteome analysis, we decided to clone the gene for GL18769, and express and purify the protein in *E*. *coli*, to test whether the protein indeed had immunomodulatory activity.

**Fig 7:**
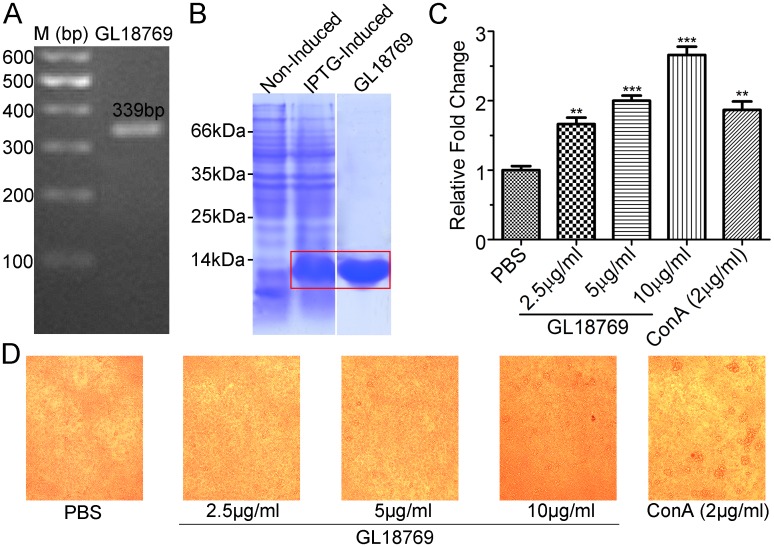
Cloning and bioactivity determination of the GL18769 protein. (A) The PCR product of GL18769 coding sequence. Total RNA was isolated from *G*. *lucidum* fruiting bodies (60 days) and amplified by reverse transcription-PCR. The GL18769 coding sequence (339bp) was amplified by PCR. (B) Expression and purification of GL18769 protein. A strong protein band appeared at approximately 13 kDa after IPTG-induction for 4 h compared with non-induction. This protein was purified using a His Trap FF column as indicated in the SDS-PAGE. (C) Stimulatory effect of GL18769 on mouse spleen lymphocytes. Compared to the ConA (2 μg/ml) treatment, GL18769 could significantly enhance the proliferation of mouse spleen lymphocytes (MSLs) in a dose-dependent manner (2.5, 5 and 10 μg/ml). (D) Representative morphology images of the MSLs treated with different dose of GL18769 (2.5, 5 and 10 μg/ml) after 36 hours. The results are means ± S.E.M. (n = 3); * P<0.05, ** P<0.01, *** P<0.001.

Because 90dF is highly lignified, it was difficult to extract intact RNA from the 90dF; therefore, we cloned the putative *GL18769* coding sequence using a total cDNA library from *G*. *lucidum* 60dF (Fig. 7A), and determined that the sequence of PCR product was identical with that of *GL18769*. The PCR product was cloned into a pET-28a vector with a His-tag at its C-terminus to produce the recombinant protein. After separating the induced bacterial lysates by SDS-PAGE, a strong band of approximately 13 kDa corresponding to the recombinant GL18769 protein was produced (Fig. 7B). The supernatant of the lysate was loaded onto a His Trap FF column from which the recombinant GL18769 protein was eluted to produce a single band of 13 kDa on SDS-PAGE (Fig. 7B). Unlike GL18769 described above, GL18770 is essentially the same as LZ-8, whose immunoregulatory activities have already been studied in some detail. We therefore did not pursue a functional analysis of GL18770 in this study.

### Determination of immunomodulatory activity of GL18769 protein

To determine whether GL18769 protein possess any immunomodulatory activity similar to other immunomodulatory proteins [49, 81], a system of mouse splenic lymphocytes (MSLs) was employed with Concanavalin A (ConA, 2 μg/ml) as the positive control. Compared to the ConA protein, GL18769 indeed stimulated the MSLs proliferation in a dose-dependent manner (Fig. 7C). Incubated at 2.5 μg/ml for 36 hours, GL18769 had a stimulating effect similar to that of 2 μg/ml ConA. Incubated at 10 μg/ml for 36 hours, GL18769 significantly stimulated the lymphocyte proliferation 2.7-fold relative to the negative control (PBS treatment). Imaging analysis by microscopy showed that GL18769 treatment induced an increase in the cell density of splenic lymphocytes, but unlike with ConA treatment, did not cause cell aggregation (Fig. 7D). This difference in effect between ConA and GL18769 implies that GL18769 might involve a lymphocyte stimulation mechanism different from that of ConA.

## Conclusions

In this study, we provided the first comprehensive attempt to elucidate the *G*. *lucidum* proteome. To date, only a limited number of studies on lignocellulolytic enzymes have been conducted in *G*. *lucidum* [51, 82, 83]. By searching against the *G*. *lucidum* genome, 61 proteins were identified as wood degrading enzymes, greatly broadening the database of lignocellulose degrading proteins of *G*. *lucidum*. Most of these enzymes (49) were identified in the fruiting bodies at 90 days. The differences in developmental stages, methods used to extract proteins, and culture conditions of *G*. *lucidum* may explain this result. Many TCA-cycle related enzymes and peptidases were detected in our proteome, suggesting their importance in the development of *G*. *lucidum*. For the first time, two argonaute-like proteins that participate in the expression of miRNA-like RNAs in fungi [75] were identified from *G*. *lucidum*. This finding implies that the miRNA-like RNAs may exist in *G*. *lucidum*.

In keeping with previous proteomic studies in other fungi, most proteins in the *G*. *lucidum* proteome were grouped into the COG categories ‘Carbohydrate transport and metabolism’, ‘Amino acid transport and metabolism’ and ‘Energy production and conversion’ [35, 40, 42]. Perhaps proteins in these COG classifications are particularly important for fungal development in general.

GL18770 is the LZ-8 protein. A small difference exists between GL18770 and LZ-8, but the difference is likely attributable to the use of different *Ganoderma* strains in the published genome report [19] and the current proteome study. Based on the current proteome data and the published genome, the GL18769 was successfully cloned, expressed and proved to be a new immunomodulatory protein. Differently than ConA, which induced aggregation and proliferation of MSLs, GL18769 increased the density of splenic lymphocytes with little cell aggregation observed. Further study should be done in the future to elucidate the mechanism for stimulating the MSLs proliferation.

*G*. *lucidum* is a macrofungus which undergoes tremendous changes from mycelium to mature fruiting body (Fig. 1). The high concentration of interfering compounds (e.g., lignin, pigments, polysaccharides, and terpenoids) makes it technically difficult to use the same protein extraction method for all the different developmental stages, especially the 90dF stage, which is highly lignified. Different methods have been tried to extract proteins from 90dF, and the method used in this study offers the best results. For mycelium and 60dF, it is easy to obtain proteins using the same TCA/acetone method. Performing a quantitative proteomic analysis for samples prepared by different methods is unreasonable; therefore, it is currently impractical to perform a quantitative proteomic study of the three developmental stages for *G*. *lucidum*. However, in this study, the qualitative proteomic analysis of this macrofungus is still significant. Further quantitative proteomic study is required to characterize *G*. *lucidum* extensively.

## Supporting Information

S1 Table Detailed information of proteins identified by LC-MS/MS searching against the *G*. *lucidum* genome database. By searching against the *G*. *lucidum* genome database, 803 proteins from the three developmental stages of *G*. *lucidum* (16 days mycelium, 60dF and 90dF) were identified by LC-MS/MS. 247, 401 and 273 proteins were identified from these three developmental stages, respectively.
(XLSX)

S2 Table Information of proteins annotated by gene onotology (GO). GO annotations for 470 proteins were classified into 39 functional groups.
(XLSX)

S3 Table Information of proteins annotated by COG. COG annotations for 357 proteins were classified into 22 sub-categories.
(XLSX)
